# Combined COVID-19 vaccination and hepatitis C virus screening intervention in marginalised populations in Spain

**DOI:** 10.1038/s43856-023-00292-y

**Published:** 2023-05-12

**Authors:** Jeffrey V. Lazarus, Marcela Villota-Rivas, Pablo Ryan, Maria Buti, Lara Grau-López, Guillermo Cuevas, José Luis Espada, William Morón, Raul Felipe Palma-Álvarez, Jordan J. Feld, Jorge Valencia

**Affiliations:** 1 Barcelona Institute for Global Health (ISGlobal), Hospital Clínic, University of Barcelona, Barcelona, Spain; 2 Faculty of Medicine and Health Sciences, University of Barcelona, Barcelona, Spain; 3 CUNY Graduate School of Public Health and Health Policy, New York, NY USA; 4 Department of Internal Medicine, Hospital Universitario Infanta Leonor, Madrid, Spain; 5 Faculty of Medicine, Complutense University of Madrid, Madrid, Spain; 6 Centro de Investigación Biomédica en Red en Enfermedades Infecciosas (CIBERINFEC), Madrid, Spain; 7 Liver Unit, Hospital Universitario Vall d’Hebron, Barcelona, Spain; 8 CIBERhd, Instituto de Salud Carlos III, Madrid, Spain; 9 Department of Psychiatry, Addiction and Dual Diagnosis Section, Hospital Universitari Vall d’Hebron, Barcelona, Spain; 10 Psychiatry Group, Mental Health and Addiction, Vall d’Hebron Research Institute (VHIR), Barcelona, Spain; 11 Biomedical Network Research Centre on Mental Health (CIBERSAM), Madrid, Spain; 12 Department of Psychiatry and Forensic Medicine, Autonomous University of Barcelona, Barcelona, Spain; 13 Harm reduction Unit “SMASD”, Madrid, Spain; 14 Toronto Centre for Liver Disease, University Health Network, Toronto, Canada; 15 Mobile testing unit, Madrid, Spain

**Keywords:** Public health, Hepatitis C, HIV infections, Health services

## Abstract

**Background:**

COVID-19 has hindered hepatitis C virus (HCV) and HIV screening, particularly in marginalised groups, who have some of the highest rates of these conditions and lowest rates of COVID-19 vaccination. We assessed the acceptability of combining HCV testing with COVID-19 vaccination in a centre for addiction services (CAS) in Barcelona and a mobile testing unit (MTU) in Madrid, Spain.

**Methods:**

From 28/09/2021 to 30/06/2022, 187 adults from marginalised populations were offered HCV antibody (Ab) testing along with COVID-19 vaccination. If HCV Ab+, they were tested for HCV-RNA. MTU participants were also screened for HIV. HCV-RNA+ and HIV+ participants were offered treatment. Data were analysed descriptively.

**Results:**

Findings show how of the 86 CAS participants: 80 (93%) had been previously vaccinated for COVID-19, of whom 72 (90%) had the full first round schedule; none had a COVID-19 vaccine booster and all received a COVID*-*19 vaccine; 54 (62.8%) were tested for HCV Ab, of whom 17 (31.5%) were positive, of whom all were tested for HCV-RNA and none were positive. Of the 101 MTU participants: none had been vaccinated for COVID-19 and all received a COVID-19 vaccine; all were tested for HCV Ab and HIV and 15 (14.9%) and 9 (8.9%) were positive, respectively; of those HCV Ab+, 9 (60%) were HCV-RNA+, of whom 8 (88.9%) have started treatment; 5 (55.6%) of those HIV+ had abandoned antiretroviral therapy, of whom 3 (60%) have re-started it.

**Conclusions:**

The intervention was accepted by 54 (62.8%) CAS participants and all MTU participants and can be used in marginalised communities.

## Plain language summary

The COVID-19 pandemic has reduced the numbers of people being screened to determine whether they are infected with the hepatitis C virus (HCV) or HIV. This is particularly the case for marginalised populations, which include people with substance use disorders (e.g., injecting drug use), those who are experiencing homelessness, and those with mental health disorders. This study explored whether these populations were willing to be tested for HCV after receiving a COVID-19 vaccination in a centre for addiction services in Barcelona and a mobile testing unit (MTU) in Madrid, Spain. Those attending the MTU were also screened for HIV. Most participants were both vaccinated and tested for HCV and HIV, as applicable, when offered. Applying this approach more widely could improve healthcare reach among marginalised populations.

## Abstract (web summary)

Lazarus et al. offered hepatitis C virus (HCV) and HIV screening, alongside COVID-19 vaccination, to adults from marginalised populations. The combined intervention was accepted by over half of participants, identifying those who needed, and were offered, COVID-19 vaccination and HCV and HIV treatment.

## Introduction

The hepatitis C virus (HCV) and HIV represent a substantial disease burden. Globally, an estimated 58 (95% confidence interval: 46–76) million people have chronic HCV^1^, resulting in 290,000 (230,000–580,000) deaths annually, primarily from cirrhosis and liver cancer^2^. In Spain, roughly 0.22% (0.12–0.32%) of the general population (20–80 years old) have an active HCV infection^3^. In the autonomous communities of Madrid and Catalonia (where the cities of Madrid and Barcelona are located, respectively), the prevalence of HCV is estimated at 1.14^4^ and 0.47^5^ cases per 100,00 inhabitants, respectively. There are approximately 38.4 (33.9–43.8) million people living with HIV globally, with the condition claiming 650,000 (510,000–860,000) lives yearly^6^. Spain has an estimated 160,000 (130,000–170,000) people living with HIV over the age of 15^7^. In the autonomous community of Madrid, the prevalence of HIV is estimated at 334.5 cases per 100,00 inhabitants^8^. Furthermore, about 2.3 (interquartile range: 1.3–4.4) million people worldwide have an HCV and HIV co-infection, of which 1.2 (0.9–1.4) million are individuals with substance use disorders (SUDs)^9^. In Spain, an estimated 3.7% (95% confidence interval: 2.9–4.7%) of HIV+ people have an HCV co-infection, as per a 2020 report from the Ministry of Health^3^.

Direct-acting antivirals can cure >95% of HCV+ people^10^. Thanks to antiretroviral therapy, the life expectancy of people living with HIV now nears that of the general population when initiated early enough^11^. However, a major barrier in treating HCV and HIV is that about 80% of people living with HCV and 15% of people living with HIV remain undiagnosed and untreated^6,10,12^. According to the Spanish Ministry of Health, around 30% of the population with SUDs infected with HCV are not aware of their status^3^ and 49.8% of new HIV cases are classified as late diagnoses^13^, making screening and treatment programmes crucial.

The COVID-19 pandemic also represents a major burden of disease, having claimed 6.8 million lives worldwide, with Spain being disproportionately affected, officially accounting for about 1.8% of all reported COVID-19 related deaths, despite representing only 0.6% of the world’s population^14^. Although 92.8% of the population (13 years or older) in Spain has received at least one dose of the COVID-19 vaccine, including 92.1% and 91.9% in the autonomous communities of Madrid and Catalonia, respectively^15^, reaching marginalised groups for COVID-19 vaccination continues to be a challenge^16^.

Marginalised populations, including people with SUDs, experiencing homelessness, and with mental health disorders, are at an increased risk for COVID-19, experience higher mortality and disease severity^17,18^, and have lower COVID-19 vaccination rates^19^. For many marginalised individuals, their main connection to healthcare is situated within community and social-based services and outreach programmes^10^. Such infrastructure provides a unique opportunity to combine COVID-19 vaccination with testing and treatment of HCV and HIV. These marginalised communities have an especially high burden of HCV^1,10,20–23^ and HIV^1,7,10,20,24^, and efforts to reach them are crucial to achieve the World Health Organization (WHO) goal of eliminating the viral hepatitis and AIDS epidemics as public health threats by 2030^10^.

COVID-19 prevention measures, such as mobility restrictions and physical distancing, have hampered HCV and HIV testing and treatment^7,13,25,26^. However, even without the pandemic, marginalised groups have low engagement in healthcare^1,10^. The criminalisation of drug use and sex work and discrimination and stigmatisation within the healthcare system^10^ lead to poor access to and retention in treatment. Even if engaged in care, comorbidities like SUDs and mental illness can hinder HCV^27^ and HIV^28^ management, impacting health outcomes. HCV and HIV elimination will not be attainable without efforts to improve access to screening, promote linkage to treatment, and retain individuals across the care continuum^10^.

The ongoing pandemic provides an opportunity to combine COVID-19 vaccination with HCV and HIV screening, during a single co-localised engagement. Approaches such as point-of-care testing (PoCT) can be used to identify people who have been exposed to HCV and HIV and performed during the post-vaccination observation period, with results in as short as five minutes^29^. Linkage to care can then be offered as needed, thereby maximising interactions with populations that are less engaged with conventional healthcare models.

The aim of this study was to assess the acceptability of combining HCV screening with COVID-19 vaccination in a centre for addiction services (CAS) in Barcelona and a mobile testing unit (MTU) in Madrid, Spain. Across the two sites, we found a ≥ 62.8% acceptance of the combined intervention, demonstrating that this approach can serve to reach marginalised populations with a critical vaccine and needed testing services.

## Methods

### Study design

**Fig. 1:**
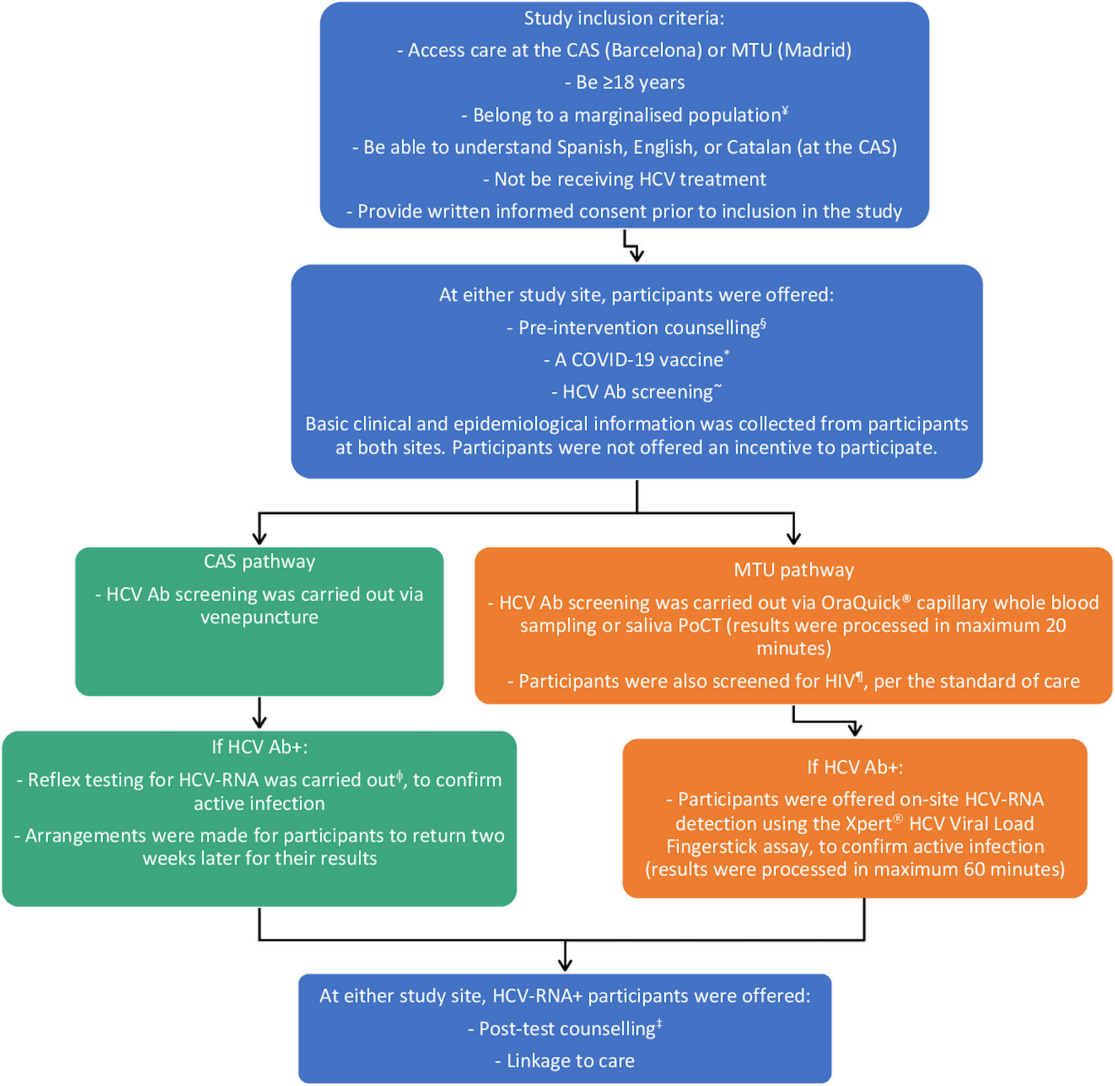
Study design. ^¥^i.e., people experiencing homelessness, those with substance use and/or mental disorders, sex workers, refugees, and undocumented migrants. ^§^Including vaccine mode of action and benefits, possible adverse reactions to vaccination, type of vaccine received, the difference between the HCV Ab versus HCV-RNA tests and what each means, follow-up steps if HCV Ab+, the impact of HCV on health, and strategies for the prevention of acquiring HCV infection. ^*^CAS participants received either a Moderna or Pfizer-BioNTech vaccine. MTU participants received a Janssen vaccine. All vaccines were provided by the public health authorities of the respective autonomous communities. ˜During the 15-minute post-vaccination observation period. ^¶^Prior to HIV testing participants were counselled on follow-up steps if HIV+, impact of HIV on health, and strategies for the prevention of acquiring HIV infection. ^†^Including the implications of being HIV+, the impact of HIV on health and benefits of treatment (assuring them that it is treatable), strategies for preventing the spread of HIV, and to contact others who may have exposed them to HIV or who they may have exposed. ^ϕ^In the microbiology laboratory where the blood sample to screen for HCV Ab had been processed. ^‡^Including the implications of being HCV-RNA+, the impact of HCV on health and benefits of treatment (assuring them that it is curable), strategies for preventing the spread of HCV, and to contact others who may have exposed them to HCV or who they may have exposed. Ab antibody, ART antiretroviral therapy, CAS centre for addiction services, HCV hepatitis C virus, MTU mobile testing unit, PoCT point-of-care testing.

This pilot study was carried out from 28/09/2021 to 30/06/2022, as per Fig. 1. All participants provided written informed consent prior to their inclusion in the study.

At the CAS, a total of four nurses, two physicians, and three administrative staff were involved in carrying out the intervention. Retainment in care at the CAS is facilitated by the fact that patients are accustomed to returning, as it is a fixed site. The MTU had four staff on site to carry out the intervention, including one physician, one nurse, and two community healthcare workers. It also uses a peer navigator system to support patients throughout their treatment process. For the purposes of this pilot, all staff were funded by the study.

The primary endpoint of this study was participants’ acceptability of HCV screening after COVID-19 vaccination, which was measured as the proportion of participants who agreed to HCV testing post COVID-19 vaccination, per site.

### Sample size

As this was a pilot study, no statistical sample size estimation was calculated.

### Variables

The variables collected about each participant are listed in Supplementary Table 1.

### Data management

Data were collected by a staff member and verified and managed by another, focal, researcher, a senior physician, per site, who oversaw the process, using Microsoft Excel version 16.57. All deidentified data were reviewed by another researcher and clarifications were sought as needed. Once verified, data were extracted per site (Supplementary Data 1) and analysed by two researchers.

### Statistical analyses

A descriptive analysis, including counts and proportions, of the variables collected from participants (Supplementary Table 1) was carried out, per site. For the CAS, the analysis was based on history of being HCV antibody (Ab) positive or negative and for the MTU on HCV-RNA and HIV status. No data were excluded from the analyses. Any instances of missing data were totalled overall and per each applicable category and proportions calculated over the total number of participants per each applicable category and overall; denominators were also adjusted as applicable, with any calculations involving missing datapoints.

### Reporting summary

Further information on research design is available in the Nature Portfolio Reporting Summary linked to this article.

### Ethical considerations

This study received ethical clearance in 2021 from the Ethics Committee of the Complutense University of Madrid, Spain (identification number: MP-001/2019) and the Ethics Committee of the Vall d’Hebron Hospital of Barcelona, Spain (identification number: AG48/2018[5359]). This study conforms to international ethical standards, including the 1975 Declaration of Helsinki.

## Results

### Centre for addiction services

**Fig. 2:**
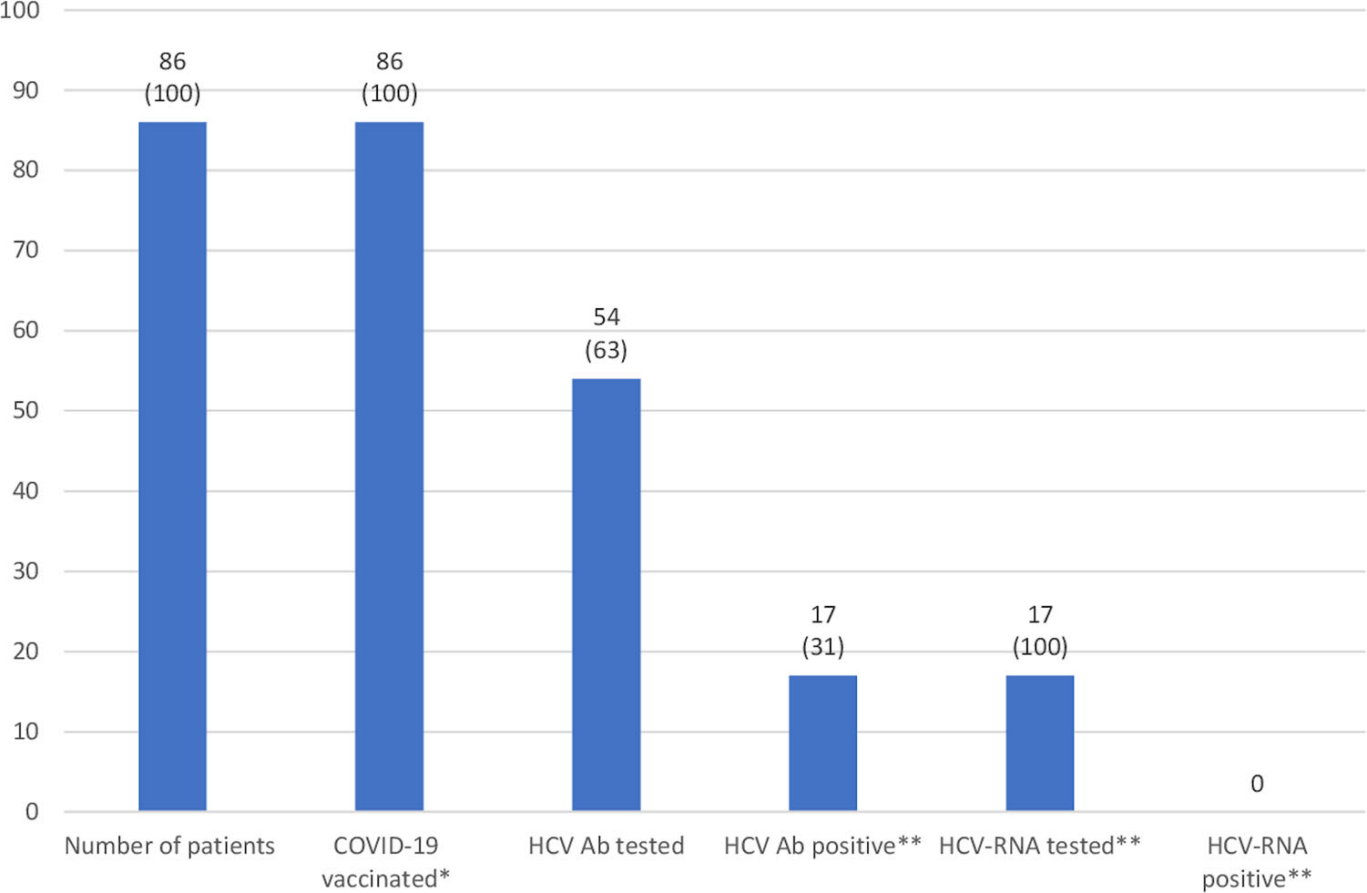
Analysis of the combined COVID-19 vaccination and HCV screening intervention at the centre for addiction services in Barcelona. Information above each bar represents *n* (%). Unless otherwise indicated, percentages are of the total *n* of participants. *Vaccinated during the study intervention. **Denominator is equal to the *n* of the prior column. *Ab* antibody, *HCV* hepatitis C virus.

Of the 86 participants (mean age 47 [standard deviation (SD): 10.1]), 66 (76.7%) were male, 73 (84.9%) were Spanish-born, all had a SUD, 16 (18.7%) had a precarious living situation or were experiencing homelessness, 4 (4.7%) had completed post-secondary education, 28 (32.6%) were unemployed, 23 (26.7%) had an incarceration history, 18 (20.9%) had mental health disorders, 10 (11.7%) had a previous sexually transmitted infection (STI) other than HIV, and 12 (14%) were HIV+ (Tables 1 and 2). Of everyone, 13 (15.1%) had a previous COVID-19 diagnosis, 80 (93%) had been previously vaccinated for COVID-19, of whom 72 (90%) had received the full first round schedule but none had received a COVID-19 vaccine booster, and all received either a Moderna or Pfizer-BioNTech COVID*-*19 vaccine during the study intervention (Fig. 2). Of all participants, 54 (62.8%) were tested for HCV Ab, of whom 17 (31.5%) were positive, of whom all were tested for HCV-RNA and none were positive. The study intervention took, on average, 23 (minimum 20; maximum 25) minutes and no adverse events, to HCV screening or COVID-19 vaccination, were identified.

**Table 1:** Sociodemographic description of participants with an HCV Ab+ or HCV Ab− history at the centre for addiction services in Barcelona.

	HCV Ab+ history	HCV Ab− history	Total
*n* (%)^a^	28 (32.6)	58 (67.4)	86 (100)
Age in years, mean (SD)	51.7 (7.5)	44.8 (10.5)	47 (10.1)
Male, *n* (%)	24 (85.7)	42 (72.4)	66 (76.7)
Spanish-born, *n* (%)	27 (96.4)	46 (79.3)	73 (84.9)
Marginalised group category, *n* (%)^b^			
Substance use disorder	28 (100)	58 (100)	86 (100)
Mental health disorder	6 (21.4)	12 (20.7)	18 (20.9)
Experiencing homelessness	2 (7.1)	2 (3.4)	4 (4.7)
Residence, *n* (%)^c^			
House/flat	20 (71.4)	48 (82.8)	68 (79.1)
Hotel/guesthouse/hostel	0	2 (3.4)	2 (2.3)
Unstable/precarious	6 (21.4)	6 (10.3)	12 (14)
Experiencing homelessness	2 (7.1)	2 (3.4)	4 (4.7)
Education level completed, *n* (%)			
Primary	23 (82.1)	28 (48.3)	51 (59.3)
Secondary	5 (17.9)	26 (44.8)	31 (36)
University (bachelor)	0	4 (6.9)	4 (4.7)
Employment, *n* (%)^c^			
Full-time (40 h/w)	1 (3.6)	12 (20.7)	13 (15.1)
Part-time (<40 h/w)	3 (10.7)	14 (24.1)	17 (19.8)
Unemployed (3–12 months)	0	4 (6.9)	4 (4.7)
Unemployed (>12 months)	10 (35.7)	14 (24.1)	24 (27.9)
Self-employed, freelance	1 (3.6)	1 (1.7)	2 (2.3)
On pension	13 (46.4)	12 (20.7)	25 (29.1)
On disability	0	1 (1.7)	1 (1.2)
History of incarceration	17 (60.7)	6 (10.3)	23 (26.7)
Tattooed, *n* (%)	24 (85.7)	22 (37.9)	46 (53.5)
Number of children, *n* (%)^d^			
1	2 (50)	5 (31.3)	7 (35)
2	0	4 (25)	4 (20)
3	0	1 (6.3)	1 (5)
4	0	1 (6.3)	1 (5)

Unless otherwise indicated, percentages are of the total *n* of participants of the corresponding HCV Ab history group.

^a^Percentage of the total *n* of participants.

^b^Numbers add up to more than the total *n* as participants could be in one or multiple categories.

^c^Percentages add up to more than 100 due to rounding.

^d^Denominator is equal to the *n* of females per HCV Ab history group.

No participant reported: being a sex worker, undocumented migrant, or refugee; having completed no education, vocational training, or a master’s degree or higher; being unemployed for <3 months; or being pregnant (females). *Ab* antibody, *h* hours, *HCV* hepatitis C virus, *SD* standard deviation, *w* week.

**Table 2:** Medical history description of participants with an HCV Ab+ or HCV Ab− history at the centre for addiction services in Barcelona.

	HCV Ab+ history	HCV Ab− history	Total
*n* (%)^a^	28 (32.6)	58 (67.4)	86 (100)
Other medical condition type, *n* (%)^b^			
Mental health disorder	6 (21.4)	12 (20.7)	18 (20.9)
Metabolic disease	0	6 (10.3)	6 (7)
Cardiovascular disease	5 (17.9)	2 (3.4)	7 (8.1)
Pulmonary disease	1 (3.6)	6 (10.3)	7 (8.1)
Other	2 (7.1)	1 (1.7)	3 (3.5)
History of COVID-19, *n* (%)	5 (17.9)	8 (13.8)	13 (15.1)
History of COVID-19 vaccination, *n* (%)	26 (92.9)	54 (93.1)	80 (93)
Number of COVID-19 vaccination doses previously received, *n* (%)^c^			
1	3 (11.5)	5 (9.3)	8 (10)
2^d^	23 (88.5)	49 (90.7)	72 (90)
COVID-19 vaccinated, *n* (%)^e^	28 (100)	58 (100)	86 (100)
History of STI, *n* (%) (other than HIV)^b^			
HBV	6 (21.4)	0	6 (7)
Moraxella	1 (3.6)	0	1 (1.2)
Syphilis	1 (3.6)	2 (3.4)	3 (3.5)
HIV+, *n* (%)	11 (39.3)	1 (1.7)	12 (14)
History of HCV treatment, *n* (%)	27 (96.4)	NA	27 (31.4)
HCV transmission route, *n* (%)			
Sexual, same sex	1 (3.6)	NA	1 (1.2)
Injecting drug use	27 (96.4)	NA	27 (31.4)
Tested for HCV Ab, *n* (%)^f^	17 (60.7)	37 (63.8)	54 (62.8)
HCV Ab+, *n* (%)^g^	17 (100)	0	17 (31.5)
Tested for HCV-RNA^g^	17 (100)	NA	17 (100)
CAS intervention length in minutes, mean (maximum; minimum)	23 (20; 25)	23 (20; 25)	23 (20; 25)

Unless otherwise indicated, percentages are of the total *n* of participants of the corresponding HCV Ab history group.

^a^Percentage of the total *n* of participants.

^b^Participants could have one or more than one other medical condition/STI other than HIV.

^c^Denominator is equal to the *n* of participants with a history of COVID-19 vaccination per the corresponding HCV Ab history group.

^d^Participants who had received one dose of the Janssen vaccine were counted as having two doses.

^e^Vaccinated during the study intervention with either a Moderna or Pfizer-BioNTech vaccine.

^f^Tested during the study intervention.

^g^Denominator is equal to the *n* of the cell above. No participant reported having contracted HCV via heterosexual sex or a blood transfusion. No participant was HCV-RNA+. Other medical conditions included cellulitis (*n* = 1) and kidney disease (*n* = 1) for the participants with an HCV Ab+ history and neuralgia and migraines for the participant with an HCV Ab− history. *Ab* antibody, *CAS* centre for addiction services, *HBV* hepatitis B virus, *HCV* hepatitis C virus, *NA* not applicable, *STI* sexually transmitted infection.

Out of everyone, 28 (32.6%) had a history of being HCV Ab+, of whom 27 (96.4%) reported that the most likely route of HCV transmission was injecting drug use and 27 (96.4%) had been previously treated for HCV. Previously HCV Ab+ participants were, on average, older than participants without a history of being HCV Ab+ (51.7 [SD: 7.5] versus (vs) 44.8 [SD: 10.5]). They were also more likely to: be male (24/28 [85.7%] vs 42/58 [72.4%]), be Spanish-born (27/28 [96.4%] vs 46/58 [79.3%]), be experiencing homelessness or have a precarious living situation (8/28 [28.5%] vs 8/58 [13.7%]), be less educated (none had completed post-secondary education vs 4/58 [6.9%]), be unemployed (10/28 [35.7%] vs 18/58 [31%]), have an incarceration history (17/28 [60.7%] vs 6/58 [10.3%]), have tattoos (24/28 [85.7%] vs 22/58 [37.9%]), have mental health disorders (6/28 [21.4%] vs 12/58 [20.7%]), have had an STI other than HIV (8/28 [28.6%] vs 2/58 [3.4%]), and be HIV+ (11/28 [39.3%] vs 1/58 [1.7%]). Additionally, they were more likely to have had a previous COVID-19 diagnosis (5/28 [17.9%] vs 8/58 [13.8%]) and less likely to accept HCV screening during the study intervention (17/28 [60.7%] vs 37/58 [63.8%]). Furthermore, out of those who had been previously vaccinated, participants with a history of being HCV Ab+ were less likely to have received the full first-round vaccination schedule against COVID-19 (23/26 [88.5%] vs 49/54 [90.7%]).

### Mobile testing unit

**Fig. 3:**
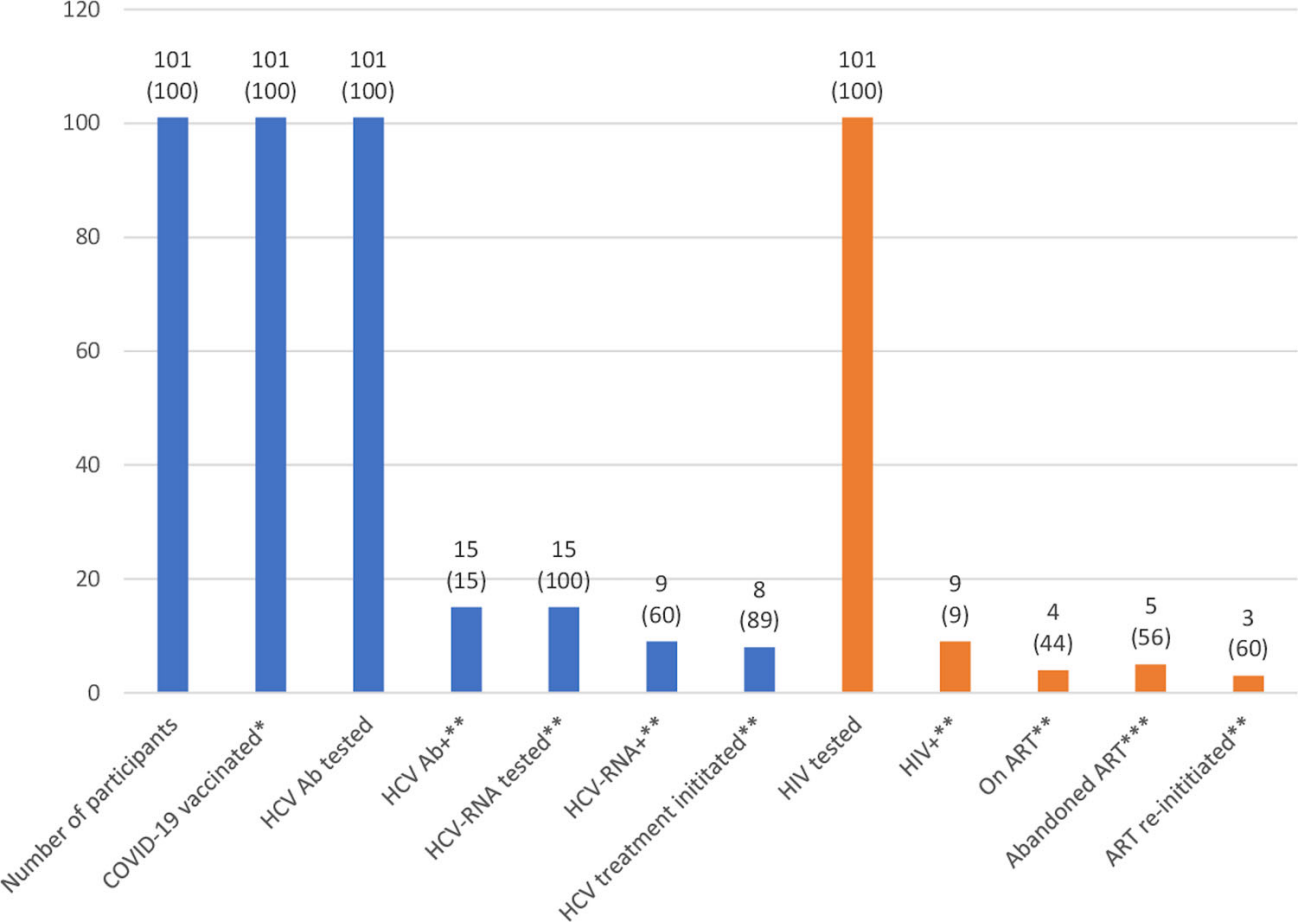
Analysis of the combined COVID-19 vaccination and HCV and HIV screening and linkage to care intervention at the mobile testing unit in Madrid. Information above each bar represents *n* (%). Unless otherwise indicated, percentages are of the total *n* of participants. *Vaccinated during the study intervention. **Denominator is equal to the *n* of the prior column. ***Denominator is equal to the *n* of HIV+ participants. *Ab* antibody, *ART* antiretroviral therapy, *HCV* hepatitis C virus.

Of the 101 participants (mean age 35.9 [SD: 11.4]), 70 (69.3%) were male, 31 (30.7%) were Spanish-born, 60 (59.4%) had a SUD, 60 (59.4%) had a precarious living situation or were experiencing homelessness, 16 (15.9%) had completed post-secondary education, 71 (70.4%) were unemployed, 29/97 (29.9%) had an incarceration history, 10 (9.9%) had mental health disorders, and 5 (5%) had a previous STI other than HIV (Tables 3 and 4). Of everyone, 12 (11.9%) had a history of being HCV Ab+, of whom 9 (75%) had been previously treated for HCV. Of all participants, 12 (11.9%) had a previous COVID-19 diagnosis, none had been previously vaccinated for COVID-19, and all received a Janssen COVID-19 vaccine during the study intervention (Fig. 3). Everyone was tested for HCV Ab and HIV and 15 (14.9%) and 9 (8.9%) were positive, respectively. Of those HCV Ab+, all were tested for HCV-RNA, of whom 9 (60%) were positive. Of those HCV-RNA+, 3 (33.3%) were HIV coinfected, 5 (55.6%) reported that the most likely route of HCV transmission was injecting drug use, 4 (44.4%) were probable reinfection cases, and 8 (88.9%) have started HCV treatment. Of those HIV+, none were new diagnoses and 5 (55.6%) had abandoned antiretroviral therapy, of whom 3 (60%) have re-started it. The average duration between positive HIV diagnosis and antiretroviral therapy re-initiation for the latter was 103 (minimum 25; maximum 138) days. The average duration between positive HCV-RNA diagnosis and treatment initiation was 83 (minimum 22; maximum 228) days. The study intervention took, on average, 33 (minimum 25; maximum 75) minutes and no adverse events, to HCV screening or COVID-19 vaccination were identified.

**Table 3:** Sociodemographic description of participants who were HCV-RNA+/HIV+, HCV-RNA+/HIV−, HCV-RNA-/HIV+, and HCV-RNA-/HIV− at the mobile testing unit in Madrid.

	HCV-RNA+/HIV+	HCV-RNA+/HIV−	HCV-RNA-/HIV+	HCV-RNA-/HIV−	Total
*n* (%)^a^	3 (3)	6 (5.9)	6 (5.9)	86 (85.1)	101 (100)
Age in years, mean (SD)	42 (16.1)	42.8 (5.7)	40.3 (8.8)	34.8 (11.9)^b^	35.9 (11.4)^b^
Missing data, *n* (%)	0	0	0	4 (4.7)	4 (4)
Male, *n* (%)	3 (100)	4 (66.7)	1 (16.7)	62 (72.1)	70 (69.3)
Spanish-born, *n* (%)	2 (66.7)	5 (83.3)	5 (83.3)	19 (22.1)	31 (30.7)
Marginalised group category, *n* (%)^c^					
Substance use disorder	3 (100)	6 (100)	6 (100)	45 (52.3)	60 (59.4)
Mental health disorder	1 (33.3)	1 (16.7)	1 (16.7)	7 (8.1)	10 (9.9)
Sex worker	0	2 (33.3)	4 (66.7)	11 (12.8)	17 (16.8)
Experiencing homelessness	1 (33.3)	6 (100)	6 (100)	28 (32.6)	41 (40.6)
Undocumented migrant	1 (33.3)	1 (16.7)	1 (16.7)	51 (59.3)	54 (53.5)
Refugee	0	0	0	15 (17.4)	15 (14.9)
Residence, *n* (%)					
House/flat	0	0	0	31 (36)	31 (30.7)
Hotel/guesthouse/hostel	0	0	0	10 (11.6)	10 (9.9)
Unstable/precarious	2 (66.7)	0	0	17 (19.8)	19 (18.8)
Experiencing homelessness	1 (33.3)	6 (100)	6 (100)	28 (32.6)	41 (40.6)
Education level completed, *n* (%)^d^					
No education	0	1 (16.7)	0	4 (4.7)	5 (5)
Primary	2 (66.7)	1 (16.7)	2 (33.3)	31 (36)	36 (35.6)
Secondary	0	4 (66.7)	4 (66.7)	36 (41.9)	44 (43.6)
University (bachelor)	1 (33.3)	0	0	9 (10.5)	10 (9.9)
Vocational training	0	0	0	5 (5.8)	5 (5)
University (≥master’s)	0	0	0	1 (1.2)	1 (1)
Employment, *n* (%)^d^					
Full-time (40 h/w)	0	0	0	11 (12.8)	11 (10.9)
Part-time (<40 h/w)	0	0	0	12 (14)	12 (11.9)
Unemployed (<3 months)	1 (33.3)	0	0	3 (3.5)	4 (4)
Unemployed (3–12 months)	0	0	0	2 (2.3)	2 (2)
Unemployed (>12 months)	2 (66.7)	6 (100)	6 (100)	51 (59.3)	65 (64.4)
Self-employed, freelance	0	0	0	4 (4.7)	4 (4)
On pension	0	0	0	3 (3.5)	3 (3)
History of incarceration	2 (66.7)	5 (83.3)	4 (66.7)	18 (22)^b^	29 (29.9)^b^
Missing data, *n* (%)	0	0	0	4 (4.7)	4 (4)
Tattooed, *n* (%)	3 (100)	4 (66.7)	5 (83.3)	43 (50)	55 (54.5)
Pregnant, *n* (%)^e^	NA	1 (50)	1 (20)	3 (12.5)	5 (16.1)
Number of children, *n* (%)^e^					
1	NA	0	1 (20)	3 (12.5)	4 (12.9)
2	NA	2 (100)	0	5 (20.8)	7 (22.6)
3	NA	0	0	3 (12.5)	3 (9.7)
4	NA	0	1 (20)	0	1 (3.2)
5	NA	0	0	1 (4.2)	1 (3.2)

Unless otherwise indicated, percentages are of the total *n* of participants of the corresponding HCV/HIV infection status category.

^a^Percentage of the total *n* of participants.

^b^Denominator adjusted due to missing data.

^c^Numbers add up to more than the total *n* as participants could be in one or multiple categories.

^d^Percentages add up to more than 100 due to rounding.

^e^Denominator is equal to the *n* of females per HCV-RNA/HIV diagnosis group. *h* hours, *HCV* hepatitis C virus, *NA* not applicable, *SD* standard deviation, *w* week.

**Table 4:** Medical history description of participants who were HCV-RNA+/HIV+, HCV-RNA+/HIV-, HCV-RNA-/HIV+, and HCV-RNA-/HIV- at the mobile testing unit in Madrid.

	HCV-RNA+/HIV+	HCV-RNA+/HIV−	HCV-RNA-/HIV+	HCV-RNA-/HIV−	Total
*n* (%)^a^	3 (3)	6 (5.9)	6 (5.9)	86 (85.1)	101 (100)
Other medical condition type, *n* (%)^b^					
Mental health disorder	1 (33.3)	1 (16.7)	1 (16.7)	7 (8.1)	10 (9.9)
Pulmonary disease	0	0	1 (16.7)	2 (2.3)	3 (3)
Thrombosis	1 (33.3)	0	0	2 (2.3)	3 (3)
Other	0	0	0	2 (2.3)	2 (2)
History of COVID-19, *n* (%)	0	0	1 (16.7)	11 (12.8)	12 (11.9)
COVID-19 vaccinated, *n* (%)^c^	3 (100)	6 (100)	6 (100)	86 (100)	101 (100)
History of STI, *n* (%) (other than HIV)^b^					
Gonorrhoea	1 (33.3)	0	0	2 (2.3)	3 (3)
Genital herpes	0	0	0	1 (1.2)	1 (1)
Syphilis	0	0	0	1 (1.2)	1 (1)
HCV Ab+ history, *n* (%)	2 (66.7)	4 (66.7)	2 (33.3)	4 (4.7)	12 (11.9)
History of HCV treatment, *n* (%)^d^	2 (100)	2 (50)	1 (50)	4 (100)	9 (75)
HCV transmission route, *n* (%)					
Injecting drug use	3 (100)	2 (33.3)	NA	NA	5 (55.6)^e^
Unknown	0	4 (66.7)	NA	NA	4 (44.4)^e^
HCV treatment initiated, *n* (%)	2 (66.7)	6 (100)	NA	NA	8 (88.9)^e^
Abandoned ART, *n* (%)	2 (66.7)	NA	3 (50)	NA	5 (55.6)^f^
ART re-initiated, *n* (%)^d^	1 (50)	NA	2 (66.7)	NA	3 (60)
MTU intervention length in minutes, mean (maximum; minimum)	75 (75; 75)	75 (75; 75)	42 (25; 75)	27 (25; 75)	33 (25; 75)
Days between HCV diagnosis and treatment initiation, mean (maximum; minimum)	137 (46; 228)	66 (22; 194)	NA	NA	83 (22; 228)
Days between HIV diagnosis and treatment re-initiation, mean (maximum; minimum)	146 (NA)	NA	82 (25; 138)	NA	103 (25; 138)

Unless otherwise indicated, percentages are of the total *n* of participants of the corresponding HCV/HIV infection status category.

^a^Percentage of the total *n* of participants.

^b^Participants could have one or more than one other medical condition/STI other than HIV.

^c^Vaccinated during the study intervention with a Janssen vaccine.

^d^Denominator is equal to the *n* of the cell above.

^e^Denominator is equal to the total *n* of HCV-RNA+ participants.

^f^Denominator is equal to the total *n* of HIV+ participants. No participant reported having: metabolic or cardiovascular disease; been vaccinated for COVID-19; or contracted HCV sexually or via a blood transfusion. Other medical conditions included gastric ulcer (*n* = 1) and hearing loss (*n* = 1). *ART* antiretroviral therapy, *HCV* hepatitis C virus, *MTU* mobile testing unit, *NA* not applicable, *STI* sexually transmitted infection.

Participants who were negative for HCV-RNA and HIV were, on average, younger compared to those positive for HCV-RNA and/or HIV (34.8 [SD: 11.9] vs 41.7 [SD: 8.8]). They were also less likely to: be Spanish-born (19/86 [22.1%] vs 12/15 [80%]), be experiencing homelessness or have a precarious living situation (45/86 [52.4%] vs all), have a SUD (45/86 [52.3%] vs all), have mental health disorders (7/86 [8.1%] vs 3/15 [20%]), be a sex worker (11/86 [12.8%] vs 6/15 [40%]), be unemployed (56/86 [65.1%] vs all), have an incarceration history (18/82 [22%] vs 11/15 [73.3%]), have tattoos (43/86 [50%] vs 12/15 [80%]), and have had an STI other than HIV (4/86 [4.7%] vs 1/15 [6.7%]). On the other hand, they were more likely to: be male (62/86 [72.1%] vs 8/15 [53.3%]), be an undocumented migrant or refugee (66/86 [76.7%] vs 3/15 [20%]), be more educated (15/86 [17.5%] had completed post-secondary education vs 1/15 [6.7%]), and have had a previous COVID-19 diagnosis (11/86 [12.8%] vs 1/15 [6.7%]). Furthermore, they were less likely to have had a previous HCV infection (4/86 [4.7%] vs 7/15 [46.7%]) and more likely to have been treated for a previous HCV infection (all vs 5/7 [71.4%]).

## Discussion

This study is one of the first to assess the acceptability of combining HCV screening with COVID-19 vaccination in marginalised communities, globally^30–32^. We found that the combined intervention was accepted by 62.8% of CAS participants and all MTU participants and was safe, as no adverse events, to either HCV screening or COVID-19 vaccination, were identified. It also optimised time use, as HCV screening was carried out during the 15-minute post-vaccination observation period, thus maximising the use of participants’ time during the intervention and preventing the need for multiple visits. Furthermore, the average intervention took only 23 minutes and the longest one 25 minutes at the CAS. As for the MTU, the average intervention was 33 minutes and the longest one 75 minutes, but the extra time allowed for participants to know by the end of the encounter whether they had an active HCV and/or HIV infection or not and be linked to care if they did.

With regards to the lower HCV screening acceptance at the CAS, even though the reasons for testing refusal were not collected, we do not consider that a lack of time drove HCV screening refusal in this study, as testing was performed during the post-vaccination observation period. We also do not think that a lack of disease knowledge played role in driving HCV testing refusal, as it did in a similar study^31^, as participants were provided with pre-intervention counselling that included information on HCV. As for factors that could have impacted HCV screening uptake, a fear of a positive result, distrust in the health system, HCV testing not being a priority for participants, and participants feeling overwhelmed post-vaccination, could be explanatory factors that have been reported in similar studies^31,32^. The methods used to screen could have also played a role, as HCV Ab testing was done via venepuncture at the CAS and PoCT at the MTU. A study of people who inject drugs found that 82.9% of participants preferred HCV Ab screening via PoCT vs venepuncture^33^. PoCT has other benefits, with a study in marginalised populations finding that participants screened via PoCT were significantly more likely to be linked to care^34^. Similarly for HIV, a systematic review found a higher likelihood of participants being linked to care via PoCT screening methods^35^. Our own findings show that screening via PoCT enables linkage to care, with 88.9% of HCV-RNA+ participants having started treatment and 60% of those HIV+ who had abandoned antiretroviral therapy having re-started it.

PoCT is encouraged by WHO to facilitate integrated, people-centred health approaches. Using a single, co-localised, healthcare encounter to provide multiple interventions, e.g., testing for multiple diseases, and, as we did, providing vaccination and linkage to care, can increase testing and treatment uptake and save costs relating to outreach, infrastructure, and human resources^10^. A programme in Lombardy, Italy, used an integrated approach similar to ours whereby they offered HCV screening to 1969–1989 birth-cohort subjects undergoing COVID-19 vaccination using PoCT and found that four (0.06%) of 7219 participants were HCV-RNA+^30^. Another programme in Salerno, Italy, offered HCV screening via PoCT to anyone (17 years or older) undergoing COVID-19 vaccination at a vaccination centre and found that one (0.05%) of 1952 participants was HCV-RNA+^31^. In our study, we found that nine (4.8%) of 187 participants were HCV-RNA+, by focusing on marginalised groups; all positive cases were found in one of the two sites (the MTU). A Canadian study that also screened for HCV post COVID-19 vaccination in a centre for addiction and mental health found that six (3.1%) of 192 marginalised individuals were HCV-RNA+^32^.

In terms of the low prevalence found in the Salerno study^31^, according to WHO, HCV screening in the general population is not considered cost-effective outside of specific settings with a high population prevalence. They thus recommend tailoring HCV screening efforts according to the epidemiology of each country. An example of this would be considering birth-cohort screening for identified birth cohorts with a higher HCV prevalence^9^. The Lombardy study used birth-cohort screening and unexpectedly found that the prevalence of HCV in the 1969-1989 birth-cohort was lower than previously estimated^30^. Regardless of the HCV prevalence found, the aforementioned studies demonstrate that co-locating COVID-19 vaccination and HCV testing and linkage to care efforts is possible.

Even screening programmes aimed at individuals considered at risk for HCV, such as people with SUDs^1,10,20–23^, may yield low HCV-RNA prevalence results, as was the case in our own study, where we found no HCV-RNA+ participants at the CAS. This finding may be explained by the fact that only 62.8% of participants accepted HCV screening and so HCV infected participants may have gone unidentified. Additionally, given that 96.4% of CAS participants with a history of being HCV Ab+ had a history of being treated for HCV and that 66.7% of MTU HCV-RNA+ participants had a history of being HCV Ab+, of which 66.7% had a history of being treated for HCV, the data suggests that the CAS is reaching more individuals previously engaged in care, vs the MTU. This is likely partly a consequence of the fixed nature of the CAS site, which facilitates retainment in care as patients are accustomed to returning, vs the transient nature of the MTU, which makes it harder for patients to return. In this regard, the MTU uses a peer navigator system to support patients throughout their treatment process, to help in mitigating this obstacle. Furthermore, a higher proportion of MTU participants had a precarious living situation or were experiencing homelessness, 59.4% vs 18.7% at the CAS, and were undocumented migrants or refugees, 68.4% vs none at the CAS, both of which are barriers to accessing healthcare. For many of these individuals, their main connection to healthcare is located within outreach programmes^10^, like those provided by the MTU. Thus, the MTU care pathway is more likely to access a higher proportion of individuals that have not engaged with healthcare previously, compared to a more conventional setting like the CAS.

The fact that there were no HCV-RNA+ participants at the CAS could also be considered an indication of a well-functioning CAS, whereby 94.1% of HCV Ab+ participants had been previously treated for HCV and cured (one participant had spontaneous HCV clearance), as none were HCV-RNA+, even though they continue to engage in high-risk behaviors like injecting drug use. An ongoing study in the Balearic Islands, Spain, is aiming to improve CAS functioning in this regard by screening and treating individuals with SUDs for HCV. Of 1050 recruited patients, 12.3% have been found to be HCV-RNA+, of whom 86% have initiated treatment, of whom 82.9% have finished it. Of the sustained virological response tests at 4 and 12 weeks performed so far, 95.7% and 94.8% showed undetectable HCV-RNA, respectively^36^. However, this model of care does not include COVID-19 vaccination and the findings from our study (≥62.8% acceptance), along with those in Canada (50.1% acceptance)^32^ and Italy (≥63.3% acceptance)^30^, demonstrate that there is a high degree of acceptability of combining COVID-19 vaccination with HCV screening. The interventions of our programme and that of the Salerno^31^ and Canadian^32^ studies also enabled 88.9%, 100%, and 66.7% of HCV-RNA+ participants to be linked to care, respectively.

Improving access to and retention in care for marginalised communities must continue to be a priority, as they are more likely to require healthcare interventions^10^. As demonstrated by our findings, marginalised groups are less likely to engage with conventional healthcare models, with no MTU participants having been vaccinated for COVID-19 and no CAS participants having received a COVID-19 vaccine booster prior to the study, despite extensive efforts in the autonomous communities of Madrid, where 92.1% of the population (13 years or older) has received at least one vaccination dose, and Catalonia, where 63.2% of the population (18 years or older) has received at least one booster vaccination dose^15^. Lower COVID-19 vaccine uptake by marginalised individuals was also found in an Australian study, where 49% of participants with a SUD had been vaccinated, while vaccination rates amongst the general population ranged from 57 to >95%^37^. Another study from the United States reported that amongst adults (18 years or older) with a SUD in San Diego, COVID-19 vaccine uptake was 9.3%, compared to >50% amongst the general adult population in the county^38^.

The reasons for COVID-19 vaccine hesitancy amongst marginalised populations have been explored previously, with a study from Australia reporting safety concerns as the most often cited reason for not wanting to undergo vaccination, amongst adults (18 years or older) with a SUD^39^. Another United States’ study based in New York found that adult participants with a SUD also expressed safety concerns, as they feared that long-term substance use may make them particularly vulnerable to vaccine side effects. This, compounded by uncertainty regarding the value of vaccination and widespread mistrust in the health system, contributed to the sample’s ambivalence towards getting vaccinated, despite acknowledgement that their SUD increased their risks from COVID-19^40^.

Tackling barriers to COVID-19 vaccine uptake specifically and healthcare engagement more generally in marginalised groups will require multifaceted approaches that address factors like mistrust, a lack of education, and misinformation^41^. During the continued roll-out of COVID-19 vaccine boosters, there is an opportunity to use resources like the Nobody Left Outside initiative checklist^42^, which is in alignment with the goals of universal health coverage and the WHO frameworks on integrated, people-centred healthcare, to promote a collaborative, evidence-based approach to service design and monitoring based on equity, non-discrimination, and community engagement. Tools of this type can be used when designing or redesigning healthcare interventions, such as outreach approaches like the MTU, which help to educate, overcome inequalities in, and improve access to healthcare for marginalised communities, by mitigating structural barriers and building trust over time via fostering of relationships with peer navigators. More conventional healthcare infrastructure, such as the CAS in Barcelona, can also be leveraged to optimise healthcare delivery for marginalised individuals by offering vaccination programmes that include education, as was done in this study. The fact that all participants accepted vaccination during this pilot demonstrates that high levels of vaccination uptake by marginalised communities are attainable.

Efforts to increase COVID-19 vaccine uptake should combine vaccination with education about and detection of other diseases like HCV^43,44^ and HIV^45^, as well as the hepatitis B virus, and linkage to care, if needed, to maximise the use of each healthcare encounter and mitigate aforementioned factors that hinder disease screening, such as a lack of knowledge and mistrust in the health system^31,32^. With regards HCV testing in particular, resources could be further maximised by omitting HCV Ab screening and only testing for HCV-RNA in individuals with a known history of HCV, as those with a previous infection will continue to test HCV Ab+^2^, which was evident in this study´s results.

Only by ensuring that healthcare is inclusive of everyone, will we achieve the WHO goal of eliminating the viral hepatitis and AIDS epidemics as public health threats by 2030^10^. As shown by our results, CAS participants with a history of being HCV-Ab+ were more likely to: be experiencing homelessness or have a precarious living situation, be less educated, be unemployed, have an incarceration history, have mental health disorders, have had an STI other than HIV, have had a previous COVID-19 diagnosis, and be HIV+. Furthermore, at the MTU, participants who were positive for HCV-RNA and/or HIV were more likely to: be experiencing homelessness or have a precarious living situation, have a SUD, have mental health disorders, be a sex worker, be less educated, be unemployed, have an incarceration history, have had an STI other than HIV, and have had a previous HCV infection; they were also less likely to have been treated for a previous HCV infection. Healthcare approaches need to be adapted to accommodate the needs of these groups.

In terms of study limitations, the generalisability of the findings is limited by our small pilot study sample size, at 187. For example, of the 86 CAS participants 32.6% had a history of being HCV Ab+ and thus this in not representative of an untested community. This fact also points to differences in existing levels of engagement between the CAS and MTU cohorts, as of the 101 MTU participants only 11.9% had a history of being HCV Ab+, which also impacts the interpretation of the results, as was considered in the discussion section. Additionally, the fact that access to PoCT for HCV and HIV is not universal limits the reproducibility of the approach used at the MTU. Furthermore, as the undertaking of a cost analysis was not part of this study, this limits the ability of the study to give context to payers about whether this might be an approach that they would consider implementing. Finally, not having collected participant’s reasons for refusing HCV screening precluded our ability to understand why testing was refused. Future studies of a similar nature should ensure that this information is collected, so that barriers to screening can be addressed accordingly.

As for strengths, this study is one of the first of its kind to assess the acceptability of combining HCV screening with COVID-19 vaccination in marginalised groups^30–32^. Despite the fact that this study is similar to the aforementioned Italian^30,31^ and Canadian^32^ studies with regards to the fact that in every study participants were offered HCV screening along with COVID-19 vaccination, our study has unique features. For instance, none of those studies screened participants for HIV while ours did, which lead to us finding that a third of HCV-RNA+ participants at the MTU were HIV con-infected. This not only provides evidence of the value of co-locating HCV and HIV screening, but also enabled for participant linkage to care for multiple conditions. Furthermore, our study was the only one that made use of a MTU outreach tool, which helps to build the evidence base around the benefits of going beyond conventional healthcare approaches to reach those that are hardest to reach. Even though our sample size was small, future studies can build on this pilot study within Spain and beyond. As demonstrated by this and the aforementioned Canadian^32^ and Italian^30,31^ studies, approaches that co-locate multiple interventions into a single encounter can be used to not only offset the effects of the COVID-19 pandemic on HCV and HIV testing and treatment^7,13,25,26^ but also increase the reach of healthcare interventions within marginalised communities.

This COVID-19 vaccination and HCV testing intervention was well accepted, safe, and optimised the use of time, while serving to reach marginalised populations with a critical vaccine and needed testing services. This innovative model of care, which also linked participants to HCV and HIV care, should be considered for future healthcare intervention planning, such as when providing COVID-19 vaccine boosters.

## Supplementary information

Supplementary Information

Supplementary Data 1

Description of Additional Supplementary Files

Reporting Summary

## Data Availability

Source data for all analyses are available as Supplementary Data 1. To preserve patient anonymity, identifying information was removed from this dataset and the raw dataset for this study cannot be made publicly available. However, it can be made available with appropriate ethical approval and by contacting the corresponding author [jeffrey.lazarus@isgobal.org].
