# Supplementary material for: Combined COVID-19 vaccination and hepatitis C virus screening intervention in marginalised populations in Spain
Source: Commun Med (Lond). 2023 May 12;3:66. doi: 10.1038/s43856-023-00292-y (PMC10180614; doi:10.1038/s43856-023-00292-y)
Supplement: Supplementary file 1 — Supplementary Information [file 43856_2023_292_MOESM1_ESM.pdf]

Supplementary information

Combined COVID-19 vaccination and hepatitis C virus screening intervention in marginalised populations in Spain

Jeffrey V. Lazarus, Marcela Villota-Rivas, Pablo Ryan, Maria Buti, Lara Grau-López, Guillermo Cuevas, José Luis Espada, William Morón, Raul Felipe Palma-Álvarez, Jordan J. Feld, Jorge Valencia

Table of Contents

Supplementary Table 1. Variables collected from study participants.. 2

Supplementary Table 1. Variables collected from study participants.

Variables	Possible answers
Date of birth	Date of birth
Country of birth	Country of birth
Gender	Male<br>Female<br>Transgender
If female, pregnancy status	No<br>Yes<br>Unsure
If female, having children status	No<br>Yes
If female and applicable, number of children	Number of children
Having a tattoo status	No<br>Yes
Incarceration history	No<br>Yes
Previous COVID-19 diagnosis	No<br>Yes<br>Unsure
If applicable, COVID-19 diagnosis date	COVID-19 diagnosis date
Previous diagnosis of a sexually transmitted infection (STI) other than HIV	No<br>Yes<br>Unsure
If applicable, report type of STI and date of diagnosis	Type of STI and date of diagnosis
Previous hepatitis C virus antibody (HCV Ab) diagnosis	No<br>Yes<br>Unsure
If applicable, HCV Ab diagnosis date	HCV Ab diagnosis date
Previous HCV-RNA diagnosis	No<br>Yes<br>Unsure
If applicable, HCV-RNA diagnosis date	HCV-RNA diagnosis date
If applicable, previous HCV treatment	No<br>Yes<br>Unsure
If applicable, HCV treatment date	HCV treatment date
HCV Ab+	No<br>Yes<br>Test not done
If applicable, HCV Ab test type and detection date	HCV Ab test type and detection date
HCV-RNA+	No<br>Yes<br>Test not done

If applicable, HCV-RNA: test type, detection and notification to participant date, and quantification and linkage to HCV care date	HCV-RNA: test type, detection and notification to participant date, and quantification and linkage to HCV care date
If applicable, HCV treatment initiation	No<br>Yes
HIV+ status	No<br>Yes<br>Unsure-if at the centre for addiction services
If applicable, antiretroviral therapy (ART) status-if at the mobile testing unit (MTU)	No<br>Yes<br>Unsure
If applicable, ART re-initiation-if at the MTU	No<br>Yes
If applicable, ART re-initiation date	ART re-initiation date
If applicable, HCV transmission route	Sexual-same-sex<br>Sexual-heterosexual<br>Injecting drug use<br>Blood transfusion<br>Unknown<br>Other-report
Marginalised group category	Substance use disorder<br>Mental health disorder<br>Sex worker<br>Experiencing homelessness<br>Undocumented migrant<br>Refugee<br>Other-report
Education level completed	None<br>Primary<br>Secondary<br>University (bachelor)<br>Vocational training<br>University (master's degree or higher)<br>Other-report
Place of residence	House or flat<br>Hotel, guesthouse, or hostel<br>Unstable or precarious housing<br>Experiencing homelessness<br>Other-report
Employment status	Full-time, 40 hours per week<br>Part-time, <40 hours per week<br>Unemployed, <3 months<br>Unemployed, 3-12 months<br>Unemployed, >12 months<br>Self-employed or freelance

	Other-disability, pensioner, etc.-report
Other medical condition	No<br>Yes<br>Unsure
If applicable, other medical condition type	Mental health issue<br>Metabolic issue, e.g., diabetes<br>Cardiovascular issue, e.g., hypertension<br>Pulmonary disease<br>Other-report
COVID-19 previous vaccination	No<br>Yes<br>Unsure
If applicable, previous COVID-19 vaccination date, type, and dosage	COVID-19 vaccination date, type, and dosage
COVID-19 vaccine applied	No<br>Yes
If applicable, COVID-19 vaccination date, type, and dosage	COVID-19 vaccination date, type, and dosage
Intervention duration (minutes)	Intervention duration (minutes)
