# Supplementary material for: Combined COVID-19 vaccination and hepatitis C virus screening intervention in marginalised populations in Spain
Source: Commun Med (Lond). 2023 May 12;3:66. doi: 10.1038/s43856-023-00292-y (PMC10180614; doi:10.1038/s43856-023-00292-y)
Supplement: Supplementary file 3 — Description of Additional Supplementary Files [file 43856_2023_292_MOESM3_ESM.pdf]

Description of Additional Supplementary Files

File Name: Supplementary Data 1

Description: Source data for study analyses. File includes data codes and data for the centre for addiction services and the mobile testing unit
