# Supplementary material for: Combined COVID-19 vaccination and hepatitis C virus screening intervention in marginalised populations in Spain
Source: Commun Med (Lond). 2023 May 12;3:66. doi: 10.1038/s43856-023-00292-y (PMC10180614; doi:10.1038/s43856-023-00292-y)
Supplement: Supplementary file 4 — Reporting Summary [file 43856_2023_292_MOESM4_ESM.pdf]

nature portfolio

Corresponding author(s): Jeffrey V Lazarus

Last updated by author(s): Mar 15, 2023

Reporting Summary

Nature Portfolio wishes to improve the reproducibility of the work that we publish. This form provides structure for consistency and transparency in reporting. For further information on Nature Portfolio policies, see our Editorial Policies and the Editorial Policy Checklist.

Statistics

For all statistical analyses, confirm that the following items are present in the figure legend, table legend, main text, or Methods section.

n/a	Confirmed	
☐	☒	The exact sample size (*n*) for each experimental group/condition, given as a discrete number and unit of measurement
☐	☒	A statement on whether measurements were taken from distinct samples or whether the same sample was measured repeatedly
☒	☐	The statistical test(s) used AND whether they are one- or two-sided<br>*Only common tests should be described solely by name; describe more complex techniques in the Methods section.*
☒	☐	A description of all covariates tested
☒	☐	A description of any assumptions or corrections, such as tests of normality and adjustment for multiple comparisons
☐	☒	A full description of the statistical parameters including central tendency (e.g. means) or other basic estimates (e.g. regression coefficient) AND variation (e.g. standard deviation) or associated estimates of uncertainty (e.g. confidence intervals)
☒	☐	For null hypothesis testing, the test statistic (e.g. *F*, *t*, *r*) with confidence intervals, effect sizes, degrees of freedom and *P* value noted<br>*Give P values as exact values whenever suitable.*
☒	☐	For Bayesian analysis, information on the choice of priors and Markov chain Monte Carlo settings
☒	☐	For hierarchical and complex designs, identification of the appropriate level for tests and full reporting of outcomes
☒	☐	Estimates of effect sizes (e.g. Cohen's *d*, Pearson's *r*), indicating how they were calculated

Our web collection on statistics for biologists contains articles on many of the points above.

Software and code

Policy information about availability of computer code

Data collection	Data were collected using Microsoft Excel version 16.57.
Data analysis	Data were analysed using Microsoft Excel version 16.57.

For manuscripts utilizing custom algorithms or software that are central to the research but not yet described in published literature, software must be made available to editors and reviewers. We strongly encourage code deposition in a community repository (e.g. GitHub). See the Nature Portfolio guidelines for submitting code & software for further information.

Data

Policy information about availability of data

All manuscripts must include a data availability statement. This statement should provide the following information, where applicable:

- Accession codes, unique identifiers, or web links for publicly available datasets
- A description of any restrictions on data availability
- For clinical datasets or third party data, please ensure that the statement adheres to our policy

Source data for all analyses are available as Supplementary Data. To preserve patient anonymity, identifying information was removed from this dataset and the raw dataset for this study cannot be made publicly available. However, it can be made available with appropriate ethical approval and by contacting the corresponding author [jeffrey.lazarus@isgobal.org].

Human research participants

Policy information about studies involving human research participants and Sex and Gender in Research.

Reporting on sex and gender	Findings apply to both sexes (male and female) and male and female genders (there were no transgender participants in the study, by chance). Sex and gender were considered in the study design and were reported by each participant to the study staff collecting data. Source data includes disaggregated sex and gender data as this information was collected and consent was obtained for sharing of deidentified individual-level data. Sex and gender based analyses were performed and reported in the manuscript.
Population characteristics	There were two sites for this study, a centre for addiction services (CAS) in Barcelona and a mobile testing unit (MTU) in Madrid, Spain, and descriptive data analyses were undertaken per site. Of the 86 participants (mean age 47 [standard deviation (SD): 10.1]), 66 (76.7%) were male, 73 (84.9%) were Spanish-born, all had a SUD, 16 (18.7%) had a precarious living situation or were experiencing homelessness, four (4.7%) had completed post-secondary education, 28 (32.6%) were unemployed, 23 (26.7%) had an incarceration history, 18 (20.9%) had mental health disorders, 10 (11.7%) had a previous sexually transmitted infection (STI) other than HIV, and 12 (14%) were HIV+. Of everyone, 13 (15.1%) had a previous COVID-19 diagnosis, 80 (93%) had been previously vaccinated for COVID-19, of whom 72 (90%) had received the full first round schedule but none had received a COVID-19 vaccine booster, and all received either a Moderna or Pfizer-BioNTech COVID-19 vaccine during the study intervention. Of all participants, 54 (62.8%) were tested for HCV Ab, of whom 17 (31.5%) were positive, of whom all were tested for HCV-RNA and none were positive. Of the 101 participants (mean age 35.9 [SD: 11.4]), 70 (69.3%) were male, 31 (30.7%) Spanish-born, 60 (59.4%) had a SUD, 60 (59.4%) had a precarious living situation or were experiencing homelessness, 16 (15.9%) had completed post-secondary education, 71 (70.4%) were unemployed, 29/97 (29.9%) had an incarceration history, 10 (9.9%) had mental health disorders, and five (5%) had a previous STI other than HIV. Of everyone, 12 (11.9%) had a history of being HCV Ab+, of whom nine (75%) had been previously treated for HCV. Of all participants, 12 (11.9%) had a previous COVID-19 diagnosis, none had been previously vaccinated for COVID-19, and all received a Janssen COVID-19 vaccine during the study intervention. Everyone was tested for HCV Ab and HIV and 15 (14.9%) and nine (8.9%) were positive, respectively. Of those HCV Ab+, all were tested for HCV-RNA, of whom nine (60%) were positive. Of those HCV-RNA+, three (33.3%) were HIV coinfected, five (55.6%) reported that the most likely route of HCV transmission was injecting drug use, four (44.4%) were probable reinfection cases, and eight (88.9%) have started HCV treatment. Of those HIV+, none were new diagnoses and five (55.6%) had abandoned ART, of whom three (60%) have re-started it.
Recruitment	This was a pilot study of people receiving care through a CAS in Barcelona and an MTU in Madrid. From 28/09/2021-30/06/2022, individuals (18 years or older) from marginalised populations (i.e., people experiencing homelessness, those with substance use and/or mental disorders, sex workers, refugees, and undocumented migrants) who accessed these sites and were able to understand Spanish, Catalan (at the CAS), or English, who were not receiving HCV treatment at the time, and provided written informed consent prior to their inclusion in the study, were offered a COVID-19 vaccine and HCV Ab screening, during the 15 minute post-vaccination observation period. MTU participants were also screened for HIV, per the standard of care. As this is a convenience sampling recruitment method, collected samples may not be representative of the population of interest and thus, the results cannot be generalised to a greater population.
Ethics oversight	This study received ethical clearance in 2021 from the Ethics Committee of the Complutense University of Madrid, Spain (identification number: MP-001/2019) and the Ethics Committee of the Vall d'Hebron Hospital of Barcelona, Spain (identification number: AG48/2018[5359]). This study conforms to international ethical standards, including the 1975 Declaration of Helsinki.

Note that full information on the approval of the study protocol must also be provided in the manuscript.

Field-specific reporting

Please select the one below that is the best fit for your research. If you are not sure, read the appropriate sections before making your selection.

☒ Life sciences ☐ Behavioural & social sciences ☐ Ecological, evolutionary & environmental sciences

For a reference copy of the document with all sections, see nature.com/documents/nr-reporting-summary-flat.pdf

Life sciences study design

All studies must disclose on these points even when the disclosure is negative.

Sample size	As this was a pilot study, no statistical sample size estimation was calculated and we aimed to recruit around 100 participants per site.
Data exclusions	No data were excluded from the analyses.
Replication	Given the nature of this prospective observational study, findings cannot be reproduced.
Randomization	Given that this was not a randomized controlled trial, randomization was not relevant to this study.

Blinding	Given that this was not a randomized controlled trial, blinding was not relevant to this study.

Reporting for specific materials, systems and methods

We require information from authors about some types of materials, experimental systems and methods used in many studies. Here, indicate whether each material, system or method listed is relevant to your study. If you are not sure if a list item applies to your research, read the appropriate section before selecting a response.

Materials & experimental systems

n/a	Involved in the study
☒	☐ Antibodies
☒	☐ Eukaryotic cell lines
☒	☐ Palaeontology and archaeology
☒	☐ Animals and other organisms
☒	☐ Clinical data
☒	☐ Dual use research of concern

Methods

n/a	Involved in the study
☒	☐ ChIP-seq
☒	☐ Flow cytometry
☒	☐ MRI-based neuroimaging
